# Good Exemplars of Natural Scene Categories Elicit Clearer Patterns than Bad Exemplars but Not Greater BOLD Activity

**DOI:** 10.1371/journal.pone.0058594

**Published:** 2013-03-26

**Authors:** Ana Torralbo, Dirk B. Walther, Barry Chai, Eamon Caddigan, Li Fei-Fei, Diane M. Beck

**Affiliations:** 1 Institute of Cognitive Neuroscience, University College London, London, United Kingdom; 2 Department of Psychology, The Ohio State University, Columbus, Ohio, United States of America; 3 Department of Computer Science, Stanford University, Stanford, California, United States of America; 4 Institute for Collaborative Biotechnologies, University of California Santa Barbara, Santa Barbara, California, United States of America; 5 Beckman Institute and Psychology Department, University of Illinois, Urbana-Champaign, Illinois, United States of America; University of Leuven, Belgium

## Abstract

Within the range of images that we might categorize as a “beach”, for example, some will be more representative of that category than others. Here we first confirmed that humans could categorize “good” exemplars better than “bad” exemplars of six scene categories and then explored whether brain regions previously implicated in natural scene categorization showed a similar sensitivity to how well an image exemplifies a category. In a behavioral experiment participants were more accurate and faster at categorizing good than bad exemplars of natural scenes. In an fMRI experiment participants passively viewed blocks of good or bad exemplars from the same six categories. A multi-voxel pattern classifier trained to discriminate among category blocks showed higher decoding accuracy for good than bad exemplars in the PPA, RSC and V1. This difference in decoding accuracy cannot be explained by differences in overall BOLD signal, as average BOLD activity was either equivalent or higher for bad than good scenes in these areas. These results provide further evidence that V1, RSC and the PPA not only contain information relevant for natural scene categorization, but their activity patterns mirror the fundamentally graded nature of human categories. Analysis of the image statistics of our good and bad exemplars shows that variability in low-level features and image structure is higher among bad than good exemplars. A simulation of our neuroimaging experiment suggests that such a difference in variance could account for the observed differences in decoding accuracy. These results are consistent with both low-level models of scene categorization and models that build categories around a prototype.

## Introduction

Human observers are able to quickly and efficiently categorize briefly presented images of natural scenes [1]–[5], whether that category is defined by an object within the scene or describes a property of the whole scene. Similarly, brain measures indicate that natural scene images can evoke differential activation very early in processing [6]–[8]. However, not all natural scenes are equivalent in regard to category membership. Some images are better exemplars of their category than others. Here we explore the effects of category membership on both human behavior and fMRI brain activity.

Pioneering work in fMRI, using univariate statistical techniques, revealed that the parahippocampal place area (PPA) and the retrosplenial cortex (RSC) play a key role in processing scenes as opposed to isolated objects [9]–[11]. More recently, this work has been extended to assess the role of these regions in natural scene categorization [5], [12]–[18]. Importantly, this body of work has moved away from standard univariate statistical techniques and instead used multivariate techniques that take advantage of the pattern of activity across an area.

For example, Walther et al. [5] used multi-voxel pattern analysis to show that activity patterns in the PPA and RSC, as well as in primary visual cortex (V1) and the lateral occipital complex (LOC), can be used to distinguish between scene categories. More importantly, scene categories could not only be discriminated in PPA, RSC and LOC, but decoding in these regions mirrored behavioral measures in two ways. First, the distribution of decoding errors in these regions, unlike in V1, correlated well with the distribution of behavioral errors made by subjects performing a similar scene categorization task. Second, PPA, and not RSC, LOC or V1, showed a decrease in decoding accuracy when the scenes were presented up-down inverted; a similar drop in accuracy was observed in a related behavioral categorization task.

Here we ask whether decoding accuracy in these same regions correlates with another aspect of human categorization behavior critical to the concept of a category, that is, the degree to which an image exemplifies its category. If these regions are sensitive to actual scene categories, then they should also be sensitive to the degree to which an image denotes a particular scene category. For example, within the range of images that we might categorize as a “beach,” some are more representative of that category than others. Will the degree to which an image exemplifies the category “beach” influence decoding in visual cortex?

Such an effect could suggest a connection between the perceived category membership of a scene and its neural representation in these areas. A difference in decoding accuracy could also be due to differences in the strength of the correlation between low-level visual features and scene category. It is possible, after all, that what determines whether a particular image is a good exemplar of a category is the degree to which its features correlate with a category prototype. Thus, regardless of what mediates better decoding accuracy for good than bad exemplars, such a correlation with human judgments would further implicate these regions in the representation of scene category.

We first verified behaviorally that good exemplars (rated as such by separate observers) were categorized more accurately and quickly than bad exemplars of a category. Then, using a similar approach to Walther et al. [5], we asked whether fMRI decoding accuracy in any of the regions previously implicated in natural scene categorization (V1, LOC, RSC and PPA) showed a similar good versus bad exemplar effect.

We asked a group of observers to rate 4025 images from six natural scene categories (beaches, city streets, forests, highways, mountains and offices) for how representative the images were of their respective category. Images were then grouped into good, medium or bad exemplars of each category based on their average ratings. Another group of participants then took part in two experimental sessions: a behavioral session, in which they categorized these (briefly presented) images, and an fMRI session, in which they passively viewed the images while being scanned. Data from the fMRI session was submitted to two analyses: 1) a univariate linear regression analysis to compare the percent signal change for good and bad exemplars, and 2) multi-voxel pattern analysis to determine whether any of the regions explored contained stronger category signals for good category exemplars than bad. Finally, we analyzed the images used in the experiments in order to explore what properties might make them either good or bad exemplars of their respective natural scene categories.

## Materials and Methods

### Participants

Nine participants from the University of Illinois (5 females, mean age 31), with normal or corrected-to-normal vision, participated in the behavioral and fMRI sessions for monetary reward.

### Ethics Statement

Both experiments were approved by the Institutional Review Board of the University of Illinois and all participants gave written informed consent according to the principles of Declaration of Helsinki.

### Stimuli

**Figure 1:**
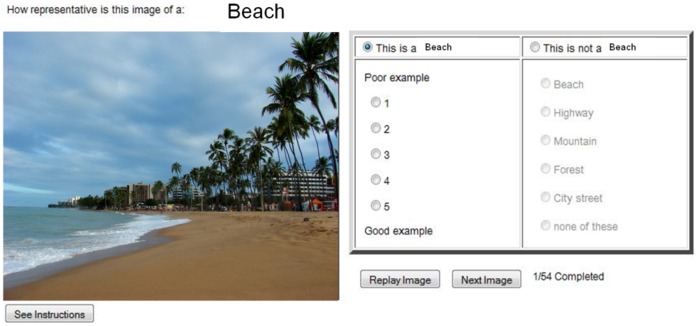
Interface used by the AMT workers to rate our images. Users could replay the image once by clicking on a button placed below the image.

4025 color images from 6 different categories (beaches, city streets, forests, highways, mountains and offices) were downloaded from the worldwide web via multiple search engines, using the six category names and their synonyms as search terms. These images were then posted to Amazon Mechanical Turk (AMT) (http://aws.amazon.com/mturk/) to be rated for how representative they were of their category. Anonymous users of the AMT web service, located all around the world, performed the task on their own computer and received $0.001 per image rated. Images were 400×300 pixels in size, but varied in the visual angle subtended across participants by the monitors and viewing distance they used. Images were shown for approximately 250 ms. Timing was controlled by the Javascript timer in the users’ web browsers, thus the actual presentation time could vary slightly depending on the users’ computer settings. Users were asked to rate each image for how good of an exemplar of a given category the image was (e.g. “How representative is this image of a BEACH?”). Responses were recorded as clicks on a graphical user interface (see Figure 1 for an example of the interface**)** and could range from 1 (“poor” ) to 5 (“good”). In order to ensure that the users of the AMT service were committed to the task, we placed a random “check” trial every 10 trials. Each check trial repeated one of the images randomly chosen among the preceding 10 trials. We computed a “discount” score for each user such that if they responded to the check trial with a different category label they received a discount of 5 and if they chose a different rating than their previous response they received a discount corresponding to the absolute value of the difference between the new and old rating. The discount score was summed over all the check trials for each user. If the total discount score exceeded 10, the data for this user was discarded. This ensured that we only retained the data of consistent and committed users. Besides being rated from 1 to 5, images could also be rated as a member of any of the other categories or as “none of them”. Images that received this response for more than one quarter of the total ratings were not used in subsequent experiments (6.36% of the total). The remaining images each received 20 ratings per image on average.

For each scene category we first selected the 80 images with the highest ratings as the candidates for “good” exemplars and the 80 images with the lowest ratings as the candidates for “bad” exemplars. In addition, we chose the 80 images closest to the midpoint between the “good” and “bad” average ratings for “medium” exemplars to be used in a preliminary staircasing procedure. We then acquired additional ratings on these candidate images to further refine our good and bad image sets. After one more round of rating, each of the 240 selected images in each category had an average of 137 ratings/image.

**Figure 2:**
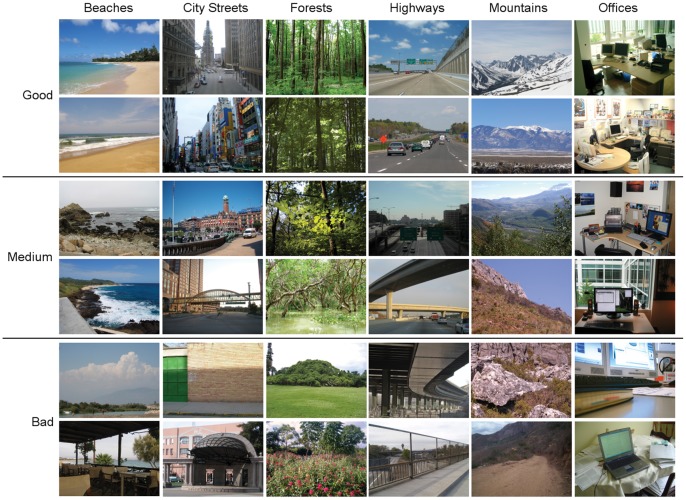
Examples of good, medium, and bad images used in the behavioral and fMRI experiments.

For each category, images from this second round of ratings were sorted in order of descending average rating. The 60 images with the highest average ratings from the 80 “good” candidates were labeled as “good” exemplars. Similarly, we selected 60 “bad” exemplars as the images with the lowest ratings. We selected 60 “medium” exemplars corresponding to the 60 central images in that ranked list. The sets of images in each rating class (good, medium, and bad) were mutually exclusive. Some examples of the images from each of the rating classes can be seen in Figure 2. Mean ratings at this stage were 4.7, 4 and 2.9 for good, medium and bad exemplars, respectively. The distribution of ratings looked similar for all 6 categories.

#### Behavioral experiment

Good, medium and bad images were scaled to 800×600 pixels. For each of the 6 categories, 60 images from each rating class were used, bringing the total number of images to 1080. Each image was presented on a CRT-monitor (resolution 1024×768, display rate 75 Hz) and subtended 22×17 degrees of visual angle. Images were centered on a 50% grey background. Stimulus presentation and response recording were controlled using the open source Vision Egg package for Python [19].

#### fMRI experiment

Stimuli were the same 360 good and 360 bad images used in the behavioral experiment (60 for each category). For four participants they were projected in a pair of MR-compatible LCD goggles (Resonance Technologies, Northridge, CA) running at a resolution of 800×600, while for the other five they were presented using back-projection set at a resolution of 800×600. In each case the images subtended 22×17 degrees of visual angle. Images were presented using the Psychophysics Toolbox for Matlab [20]–[21].

### Procedure

#### Behavioral experiment

Participants performed six-alternative forced-choice categorization of the images. The session was comprised of: a training phase during which participants learned the response mappings between the six categories and their corresponding keys; a staircasing phase, during which image presentation time varied to reach 65% classification accuracy for each individual participant; and an experimental phase during which we tested the participants’ categorization performance at his or her individual presentation time.

During each trial participants viewed a fixation cross for 500 ms prior to a brief image presentation (image duration depended on the experiment phase) that was followed by a perceptual mask (500 ms duration; see [5] for examples of the masks). Finally, a blank screen was presented for 2000 ms. Participants were asked to press one of six keys on the computer keyboard to indicate the category of the viewed image. If no input was given, the trial timed out after the 2000 ms fixation period and was excluded from the analysis.

During the training phase images were presented for 250 ms to ensure adequate learning of response mappings. Once an 80% accuracy rate was achieved in the training phase (after 60 trials on average), the QUEST algorithm [22] was used to staircase the image presentation time to achieve 65% classification accuracy for each individual participant (53 trials on average). Images from our set of medium exemplars were used for training and staircasing. Staircasing was terminated when the standard deviation of the display times over a block was less than the refresh period (13.3 ms) of the monitor. The image presentation duration obtained during the staircasing phase of the experiment was used during the testing phase of the experiment. The average presentation time across subjects was 64 ms (ranging from 26 ms to 133 ms).

During the testing phase, good and bad exemplars alternated in separate blocks of 20 images each. Participants completed a total of 36 experimental blocks. The good and bad image sets were only viewed in the testing phase of the experiment and each image was shown exactly once across the whole session. Participants received auditory feedback (800 Hz pure tone, 100 ms) for incorrect responses in both the training and staircasing phases of the experiment, but no feedback was provided in the testing phase.

#### fMRI experiment

In order to ensure high signal strength in the scanner, images were presented for longer in the fMRI experiment than in the behavioral experiment. Participants performed the fMRI experiment first. They passively viewed images for 1.6 seconds each, arranged in blocks of 10 images, with no interstimulus interval. Each run contained 6 different blocks corresponding to the 6 categories. Blocks of images were interleaved with blank periods lasting 12 seconds to allow BOLD response to return to baseline. The scanning session was comprised of a total of 12 runs, with 6 runs of good and 6 runs of bad images presented in alternating order with the starting condition (good or bad) counterbalanced across subjects. The category order was randomized but replicated for two consecutive runs (one good and one bad). Each image was shown once in the whole session.

### MRI Acquisition and Preprocessing

Scanning was performed at the Biological Imaging Center at the University of Illinois at Urbana-Champaign. T1-weighted anatomical images and gradient-echo echo planar (EPI) images were acquired in a 3T-head only scanner (Allegra, Siemens) using a standard head coil. EPI images were collected from the entire brain (TR = 2 s, TE = 30 ms, flip angle = 90, matrix 64×64; FOV 22 cm) in interleaved order. 90 volumes of 34 axial slices (3.438×3.438 mm in-plane resolution) were collected in each functional run. Slice thickness was 3 mm and gap size was 1 mm. The first 4 volumes of each run were discarded. A high resolution structural scan (1.25 mm×1.25 mm×1.25 mm; MPRAGE) was collected to assist in registering the images with the retinotopic mapping data. Functional data were motion corrected to the middle image of the 6th run, and normalized to the temporal mean of each run using AFNI [23]. For the pattern recognition analysis no other image processing steps, such as spatial smoothing, were performed.

#### Multi voxel pattern analysis

To address whether the category-specific information in various brain regions differed between good and bad images we constructed a decoder previously shown to be effective for decoding scene category from multi-voxel fMRI activity [5]. Specifically, after pre-processing, in a given ROI (see below for definition of the ROIs) we extracted the eight time points corresponding to each presentation block, shifted by 4 seconds to approximate the delay in the BOLD response. Multi-voxel pattern analysis was then performed separately on the good and bad runs. We elaborate on the procedure using good exemplars as an example, but the same procedures were used for the bad exemplars. Using five of the six “good” runs, a support vector machine (SVM) classifier with a linear kernel (C = 0.02) was trained to assign the correct category labels to patterns of fMRI activity in the ROI. The classifier was then tested on the fMRI activity from the left-out run. The classifier was trained and tested on each time point separately (as opposed to averaging activity across a block), and disagreements regarding the predicted category label within blocks were resolved by majority voting, i.e., each block was labeled with the category that was most frequently predicted among the eight volumes in the block. Ties were resolved by selecting the category with the largest SVM decision values before thresholding. The procedure was repeated six times with each of the six good runs left out in turn in a leave-one-run-out (LORO) cross validation procedure, thus generating predictions for the blocks in each run. Decoding accuracy was measured as the fraction of blocks with correct category predictions, providing an indication of the strength of the category-specific information in a given ROI for good exemplar images. The same LORO cross-validation procedure was performed for the bad image runs to arrive at the equivalent measure for bad exemplars. Significance of decoding accuracy results was established with a one-tailed t-test, comparing the mean of the accuracies over participants to chance level (1/6). A two-tailed, paired t-test was used to assess whether there was a significant difference in decoding accuracy between good and bad exemplars.

#### Univariate good/bad analysis

We performed a univariate linear regression analysis to compare the percent signal change in good and bad images. For this analysis, in addition to the pre-processing mentioned above, fMRI images were spatially smoothed (6 mm FWHM). We defined two regressors of interest: blocks of good images and blocks of bad images were modeled separately and convolved by a gamma function to approximate the hemodynamic response [24]. We performed a linear contrast between these two regressors and extracted the percent signal change in each participant’s V1, PPA and RSC ROIs. In each of the ROIs, mean percent signal change values for good and bad images were submitted to a two-tailed t-test to determine whether they differed significantly.

#### Regions of Interest

Based on previous work [5] we identified 5 separate ROIs: V1, the parahippocampal place area (PPA), the retrosplenial cortex (RSC), the lateral occipital complex (LOC) and the fusiform face area (FFA).

In a separate scanning session, V1 was delineated using standard retinotopic mapping procedures and analyses described elsewhere: a meridian mapping procedure was used for participant 3 [25] and a traveling wave procedure was used for the remaining participants [26]. LOC, FFA, PPA and RSC were identified in separate functional localizer scans, where blocks of images of faces, houses, objects, scrambled objects, landscapes and cityscapes were presented. Each block consisted of 20 images of a given category, where each image was presented for 450 ms followed by a 330 ms inter-stimulus interval. In a given run, 4 blocks of each category were shown while a fixation block of 12 seconds was interleaved every two or three blocks. Participants performed a 1-back task, pressing a button each time an image was repeated. Two functional scans (139 volumes each) were recorded in a given session. All subjects were scanned in two sessions while 1 participant required an additional session due to a weak BOLD signal in the previous scans. The 3dAllineate function in AFNI [23] was used to register the images across localizers and experimental sessions.

EPI images from the localizer runs were motion corrected, smoothed (4 mm FWHM) and normalized to the mean of each run. BOLD response in each type of block was modeled separately and convolved with a gamma variate function of the hemodynamic response [24]. ROIs were defined from linear contrasts as the sets of contiguous voxels that differentially activated in the following comparisons: PPA and RSC were identified by a (cityscapes, landscapes)>objects contrast, LOC by an objects>scrambled objects contrast and FFA was identified by a faces>(objects, cityscapes, and landscapes) contrast. For all localizer contrasts, a maximum threshold of p<2×10^−3^ (uncorrected) was applied. Stricter thresholds were used when necessary to break clusters that spanned multiple ROIs. There was no overlap between any of the ROIs, and all ROI voxels were used for the pattern analysis without any further voxel selection.

### Image Analysis

To explore whether good images are more or less variable than bad images across a given feature space we extracted features describing the form and color of the images. To create a “form” space we created grayscale versions of the images that were downsampled to 600×450 pixels and convolved them with 64 Gabor filters (8 orientation×8 frequencies) with kernel size of 8×8 pixels. As a result we obtained a 64-element vector for each pixel location with each vector entry storing the response of one Gabor filter. We averaged the 64 element vectors from all of the 600×450 pixel locations to obtain a global description of our scene image.

To capture the distribution of pixel colors over the entire image we created a “color” feature space. For this purpose, we described each image with a two dimensional histogram with the dimensions of hue and saturation discretized uniformly into 8 values, resulting in a 64 element vector (8 hues×8 saturations). The histogram was computed over the entire image, binning the color values from all of the 600×450 pixel locations to obtain a global description of our scene image.

We then estimated the variance of the images as the distance of each image from the average image in each of these feature spaces. To this end we performed singular value decomposition of the covariance matrix of the feature vectors and summed the eigenvalues of the diagonalized covariance matrix. These eigenvalues represent the amount of variance in the feature vectors along the direction of the eigenvectors of the covariance matrix. Their sum captures the overall variance present in the feature representations of the images. We performed this step separately for the good and the bad exemplars for each scene category and compared variance for each scene category as well as pooled over all categories.

### Simulation Analysis

In order to evaluate if a difference in variance among the exemplars of categories can lead to the observed differences in fMRI decoding accuracy we performed a numerical simulation of our fMRI experiment. We modeled the neural activity elicited by scene categories as multivariate Gaussian distributions with isotropic covariance 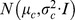. Activity for an exemplar was modeled as a random draw from this distribution. To account for the different variances between good and bad exemplars, we used smaller values of 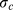
for good 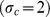
than bad 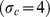 exemplars. In order for the simulation to closely follow the experiments, we estimated the mean of the multivariate Gaussian distribution for a scene category 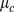 from the sample mean of the patterns of voxel activity elicited in PPA by that category for each of our eight human subjects in turn. Note that we did not model the correlations between voxels in this simulation.

We composed the time course of the simulated experiment to mirror the block design of our fMRI experiment: 12 seconds of fixation (modeled as zero neural activity) were followed by a 16-second block of images, composed of ten image activity patterns of 1.6 seconds each, randomly drawn from the same category distribution. Each category block was followed by 12 seconds of fixation, and for each block the activity was drawn from a different category distribution. We generated data for six runs, with each run containing six blocks, one from each category in a random order. The neural activity was then convolved with a Gamma function to model the hemodynamic response: 
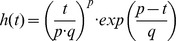
, with *p* = 8.6 and *q* = 0.547 [21]. Finally, we added normally distributed measurement noise from 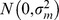. We estimated the standard deviation of the measurement noise from the residuals of the univariate regression analysis in the PPA as 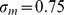. Once we had computed the simulated fMRI activity, we analyzed it with the same leave-one-run-out cross validation procedure as described for the multivoxel pattern analysis of our experimental data.

To verify that a difference in variance between good and bad images would result in poorer decoding regardless of whether we ran a blocked or event-related fMRI design, we also simulated a fast event-related experiment. For this simulation we constructed six runs with 60 trials each. Following an initial fixation period of 12 seconds duration we added activity for an image from one of the six categories for 1.6 seconds, followed by 2.4 seconds fixation before the presentation of the next image. We randomly interleaved trials for 60 exemplars (10 from each of the six categories) within a run. Including a final fixation period of 12 seconds, this resulted in a total run length of 264 seconds, compared to 192 seconds for the blocked design. Activity for the event-related design was convolved with the same hemodynamic response function (HRF) as described above, and measurement noise with the same variance was added. We then performed a regression analysis separately on each run with regressors for each of the six categories. Regressors were convolved with the same HRF as the simulated neural activity. The sets of beta-weights for the categories were used as inputs to the leave-one-run-out cross validation analysis.

We performed the block and the event-related simulations 100 times for each of the eight subjects, both for good and bad exemplars, each time with a new random draw of the exemplar activity and the measurement noise. Significance of the difference between the decoding accuracies for good and bad exemplars was computed with a two-tailed, paired t-test over eight subjects.

## Results

### Behavioral Categorization Task

**Figure 3:**
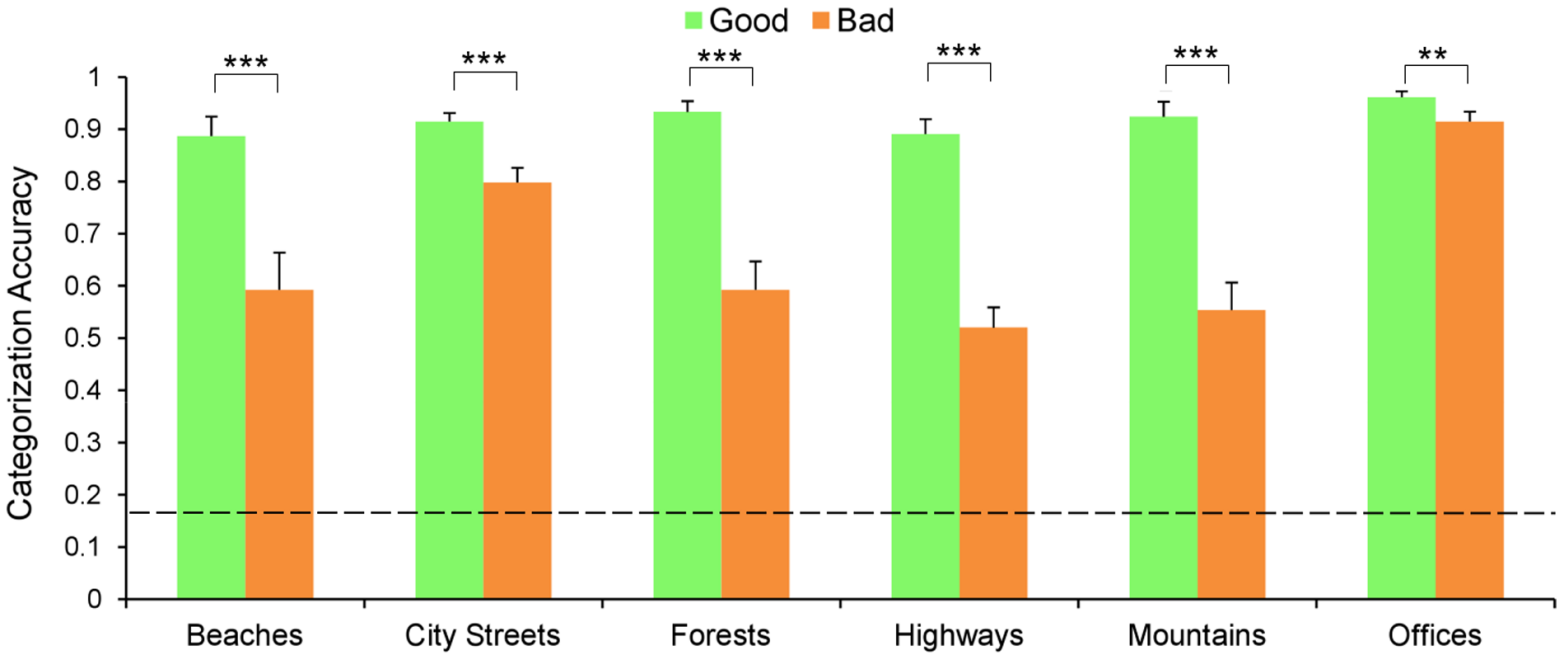
Results of the behavioral categorization task. Graph depicts categorization accuracy of good (green) and bad exemplars (orange) across categories. Error bars show standard error of the mean over nine subjects. The dashed line marks chance level (0.167). Good vs. bad comparisons are significant for all categories; **p<0.01. ***p<0.001.

Participants were significantly more accurate at categorizing the briefly presented good images than the bad images (92% and 66% respectively; t(8) = 11.57, *p*<0.001; chance was 16.67%), and this effect was significant for all six categories (see Figure 3; t(8) = 5.86, *p*<0.001 for beaches; t(8) = 5.13, *p*<0.001 for city streets; t(8) = 8.36, *p*<0.001 for forests; t(8) = 7.63, *p*<0.001 for highways; t(8) = 6.03, *p*<0.001 for mountains; and t(8) = 3.84, *p*<0.01 for offices).

Response times revealed a similar effect. Accurate responses were significantly faster for good images than bad images (892 ms and 1028 ms respectively; t(8) = 8.82, *p*<0.001), and this effect was significant across all the categories (t(8) = 8.53, *p*<0.001 for beaches; t(8) = 6.27, *p*<0.001 for forests; t(8) = 4.42, *p*<0.01 for highways; t(8) = 5.2, *p*<0.001 for mountains and t(8) = 2.58, *p*<0.05 for offices) except for city streets in which the difference was marginally significant (t(8) = 2.10, *p* = 0.068).

Moreover, to look for more fine-grained correlations between image rating and categorization accuracy we correlated these two measures separately for good and bad images. We observed a significant positive correlation between ratings and categorization accuracy for the bad images (r = .145, *p*<0.05), indicating that across the bad exemplars, images that are less representative of the category were categorized less accurately. The same correlation was not significant for the good images (r = −0.06, *p* = 0.24). However, it should be noted that the lack of correlation here may be due to substantially smaller variability in the ratings of good images (SD = 0.53) than the bad images (SD = 1.25).

### Multivariate fMRI Analysis

Having established that humans do indeed find good exemplars easier to categorize than bad exemplars of a category, we asked what effect good and bad exemplars would have on fMRI decoding rates.

Data from one participant was excluded from the fMRI analyses due to excessive movement and a low signal-to-noise ratio, and only 10 runs of fMRI data were included for another participant due to technical problems during data collection in the final 2 runs of the session. In separate functional scans (see ROIs section for details) we identified five ROIs (mean number of voxels and standard deviation in parenthesis): the PPA (93±28 voxels), the RSC (55±13 voxels), the LOC (72±32 voxels), the FFA (66±31 voxels) and V1 (229±161 voxels).

If a particular ROI is sensitive to scene category, then it should be sensitive as well to the degree to which an image denotes a particular category. Thus, we should find a difference in the decoding accuracy of good images compared to bad images. We tested for the presence of such an effect in the decoding data in the following way. We trained and tested a decoder on good images, using LORO cross validation, and compared the resulting decoding accuracy to that obtained when the decoder was trained and tested on bad images. First, when we trained and tested the decoder on good images, decoding accuracy (rate of correctly predicting the viewed scene category from the voxels’ pattern activity) was significantly above chance (16.7%) in V1 (27%, t(7) = 2.57, *p*<0.05), PPA (32%, t(7) = 4.88, *p*<0.001) and RSC (29%, t(7) = 5.60, *p*<0.001), but not in FFA and LOC (16%, *p* = 0.66 and 18%, *p* = 0.28 respectively). When we trained and tested the decoder on the bad images, decoding accuracy was significantly above chance in PPA (22%, t(7) = 2.31, *p*<0.05) but not in the other ROIs (17%, *p* = 0.53 for V1; 20% for RSC, *p* = 0.11 for RSC; 19%, *p* = 0.09 for FFA and 18%, *p* = 0.21 for LOC).

**Figure 4:**
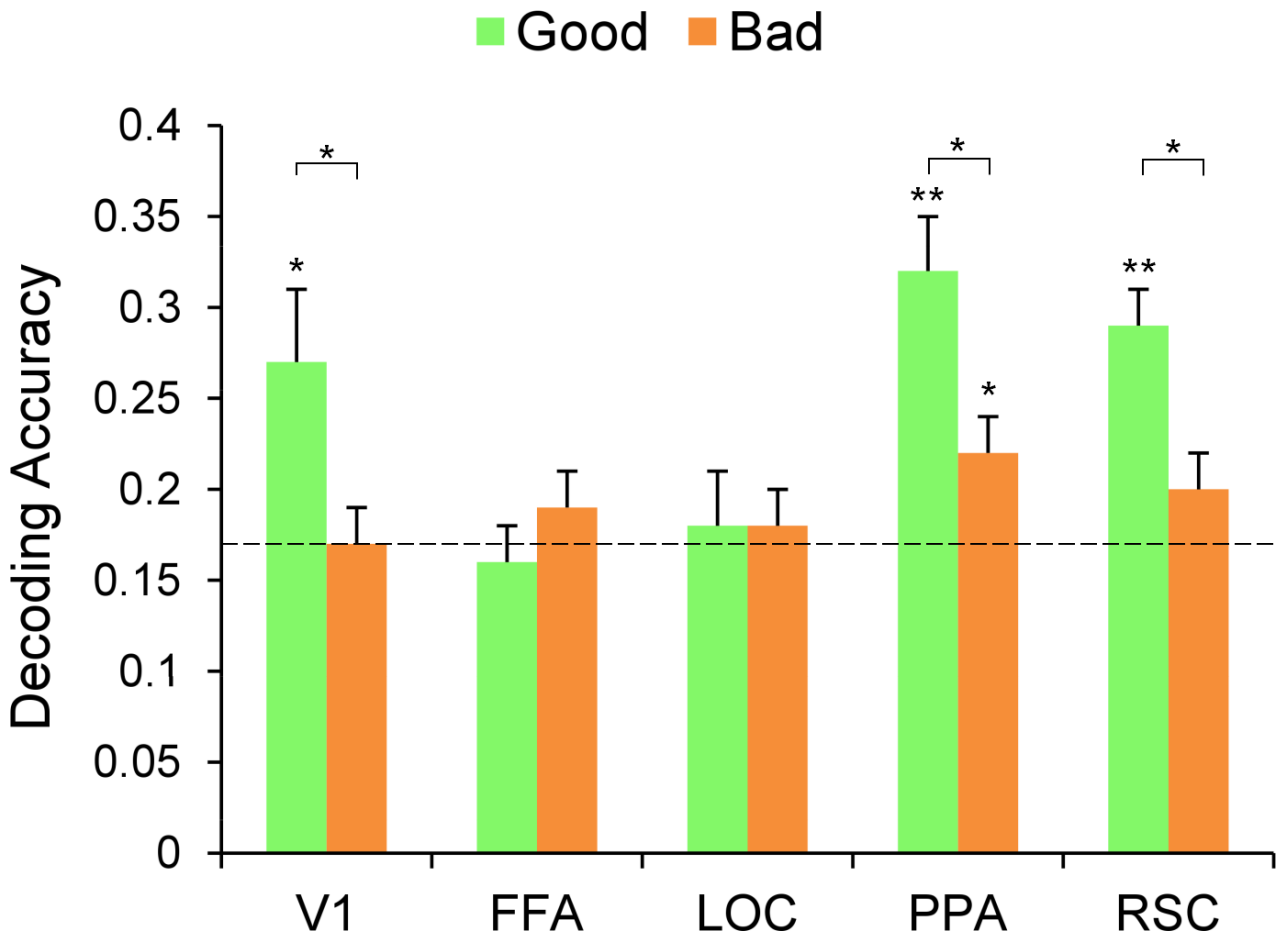
Accuracy of decoding scene category from V1, FFA, LOC, PPA and RSC. A decoder was trained and tested on fMRI activity evoked by good exemplars (green), and trained and tested on fMRI activity evoked by bad exemplars (orange). Error bars show standard error of the mean across subjects. The dotted line marks chance level (0.167). *p<0.05; **p<0.01.

**Figure 5:**
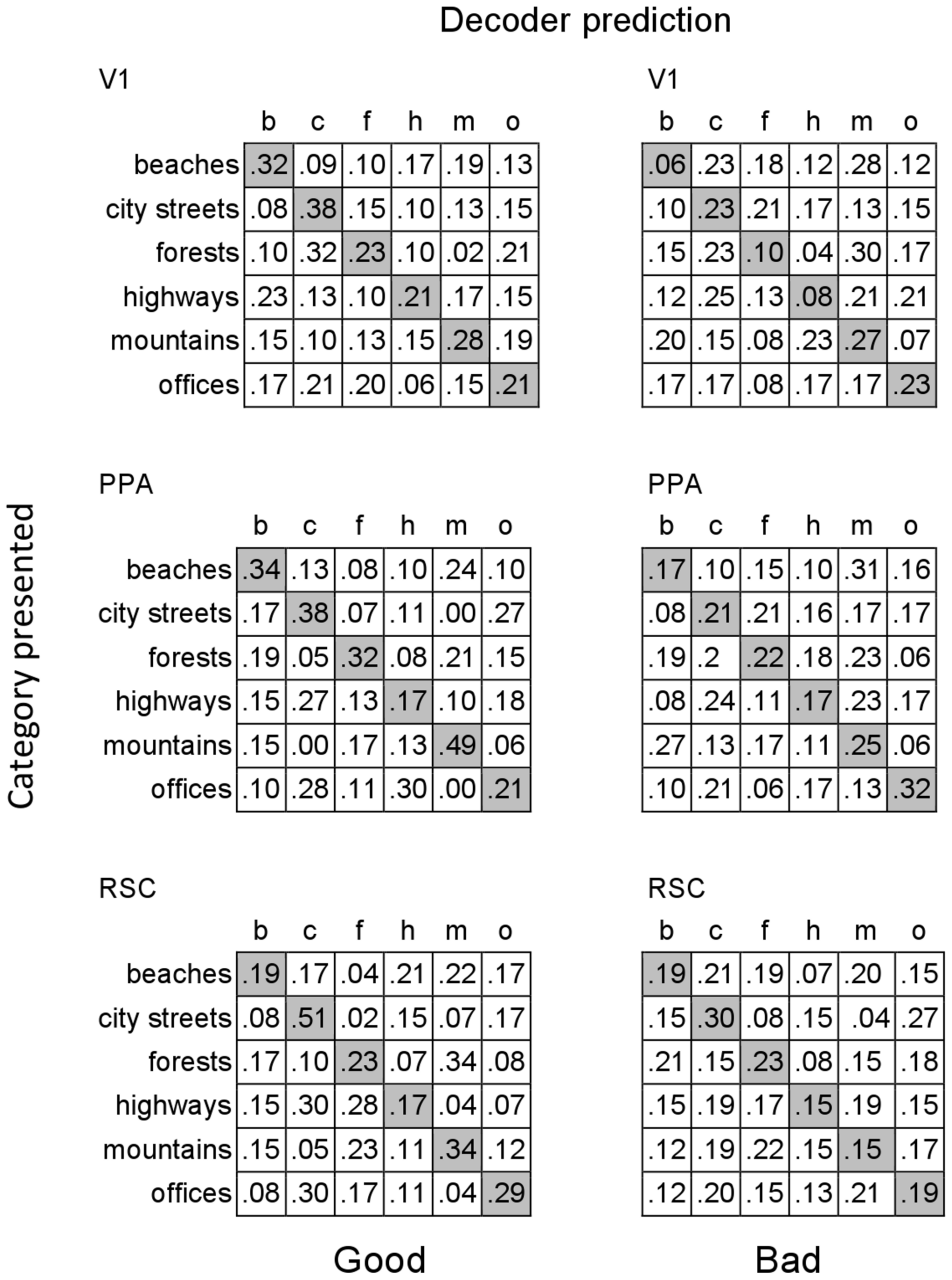
Confusion matrices for decoding of scenes categories in V1, PPA and RSC. The decoder was trained and tested on good exemplars (left column) and trained and tested on bad exemplars (right column). The rows of each matrix indicate the categories presented (ground truth) and the columns indicate the predictions of the decoder. Diagonal entries are correct decoding rates for the respective categories, and off-diagonal entries indicate decoding errors.

We looked for a good/bad effect by comparing the decoding performance for good versus bad images (2-tailed paired t-test). V1, PPA and RSC showed a significant decrement in decoding accuracy for bad exemplars relative to good exemplars: t(7) = 3.00, *p*<0.05 for V1; t(7) = 2.76, *p*<0.05 for PPA and t(7) = 2.45, *p*<0.05 for RSC. These data suggest, as predicted, that these ROIs are sensitive to category information, i.e. that category-related information in these areas is clearer for good than bad exemplars. No such effect was found in LOC and FFA (t<1 for both ROIs; see Figure 4), but this is not surprising given that decoding did not exceed chance in these regions for either good or bad exemplars. See Figure 5 for confusion matrices for V1, PPA and RSC.

Thus, in keeping with previous research [5], [14], we once again show a correlation between activity in visual cortex and human behavior: images that humans find easier to categorize are also more accurately categorized by the decoder. However, unlike previous research, this correlation extends to V1, suggesting that differences between good and bad exemplars include features encoded in V1.

### Image Analysis

**Figure 6:**
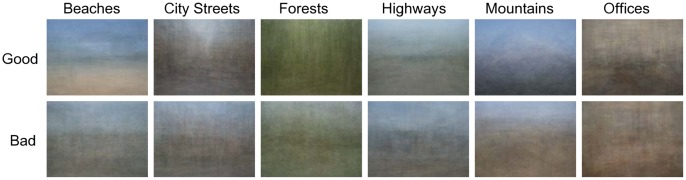
Average images. Pixel-wise RGB average images of good (first row) and bad (second row) exemplars across the categories (columns).

One possibility for the decoding advantage in both V1 and later visual areas is that good exemplars are all more similar to a particular prototype than bad exemplars. To asses whether this might be the case, we computed a pixel-wise average image of all 60 images from a category, separately for the good and bad exemplars (Figure 6). Specifically, we simply averaged the RGB values at each pixel in the image. Interestingly, the average image of the good exemplars reveals fairly clear spatial structure that makes it possible to identify the category (e.g., a mountain peak can be made out in the good mountain average). The same is not true of the pixel-wise average of the bad exemplars; little systematic structure is discernible in these images (Figure 6). This analysis not only suggests that good exemplars are more similar to each other in structure than bad exemplars, it is also suggestive of a potential prototype for each category. For instance, a prototypical mountain scene may contain a single peak in the center; a prototypical city street scene may contain a street that recedes in depth with tall buildings on either side.

**Figure 7:**
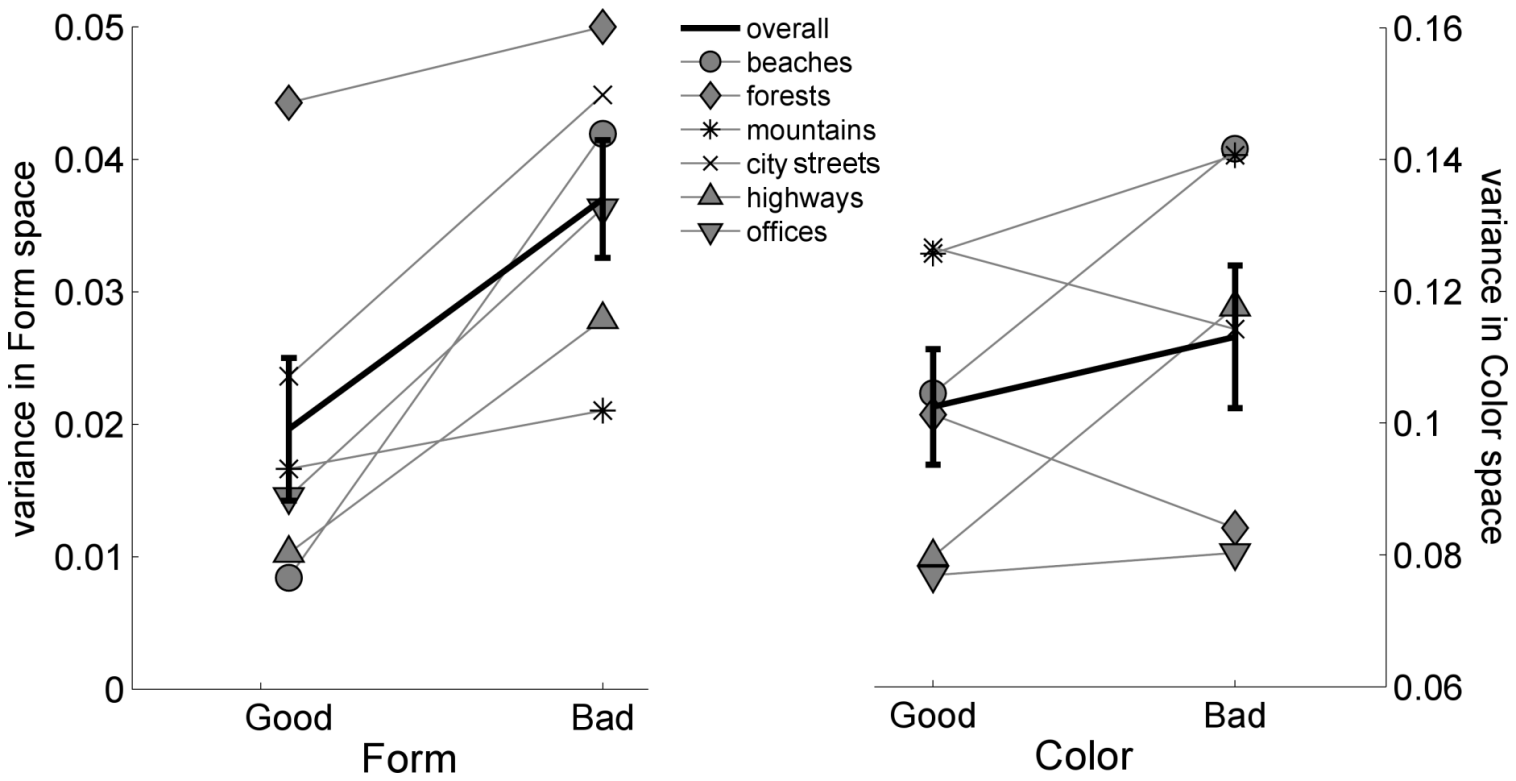
Variance across feature spaces. Variance of good and bad exemplars across form and color (left and right panels, respectively) spaces.

To further explore whether good images are in fact less variable in low-level feature space than bad images we computed how far each image is from the average image in two feature spaces. Because, as our pixel-wise average images illustrate, good exemplars appear to be distinguished from bad exemplars in the consistency of both their spatial layout and color, we chose one feature space that capture the form (or structure) of scenes and one that captured the distribution of colors across an image. Figure 7 shows the variance (mean square distance from the mean image) for each feature space. In the “form” space (using Gabor filters), the variance of the good exemplars is smaller than the variance of the bad exemplars, and this is consistent for all the categories (see the left panel of Figure 7). A similar pattern can be seen for the color space, although it is less consistent across categories: 4 out of the 6 categories have a smaller variance for good than bad exemplars (see right panel of Figure 7).

In short, analysis of the images themselves suggests that good exemplars of a category have more similar low-level image statistics to each other than bad exemplars do. We note that this similarity is itself a novel finding as our raters were asked only to rate the images for how representative they were of their category. At no point did we suggest to the raters that they match the image to a prototype or that representative images should be similar to one another. These more consistent low-level image statistics for good than bad exemplars of a category not only could contribute to the more accurate decoding of good images in the brain but it is also suggestive of the categories being organized around a prototype.

### Simulation Analysis

Is it plausible that the differences in within-category variance between good and bad exemplars gave rise to the differences in decoding accuracy that we found in the fMRI data? To address this question we performed a computer simulation of the fMRI experiment in which we manipulated the variance in the patterns of activity evoked by good and bad exemplars. We simulated the neural activity for exemplars of scene categories as multivariate Gaussian distributions around a prototype mean. So that our prototype approximated the patterns observed in our data we simulated the prototype by taking the mean activity at each voxel for each of the six scene categories in the PPA from each of the eight subjects in turn. Importantly, we used two different settings for the variance of the category distributions, low variance to simulate good exemplars and high variance to simulate bad exemplars. In accordance with the image analysis results we set the variance for bad exemplars to double the value for good exemplars.

**Figure 8:**
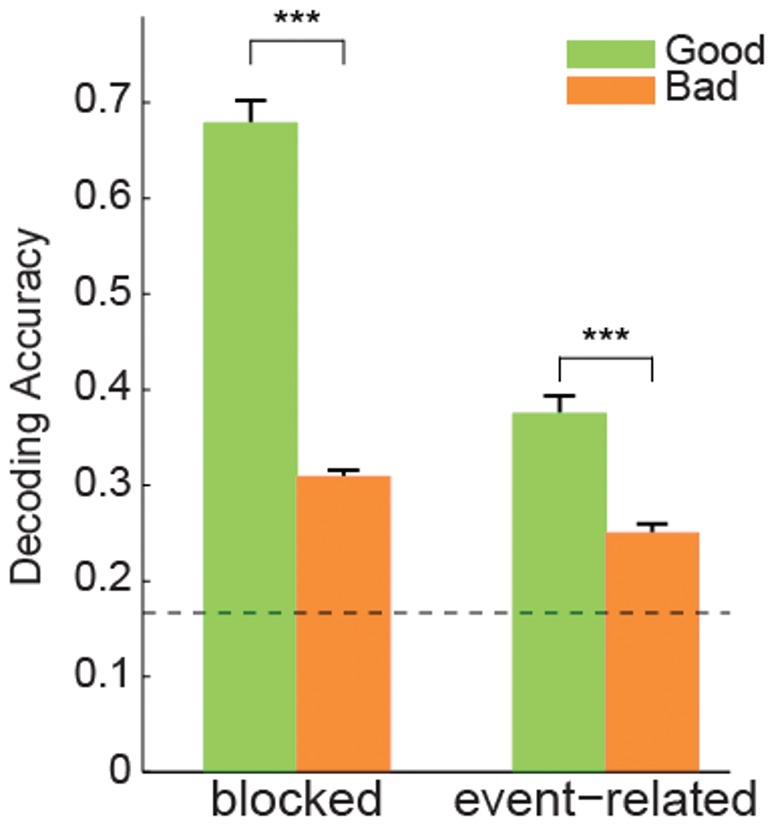
Decoding accuracy for simulated fMRI activity for good and bad exemplars. Results simulating a block design are on the left and a fast event-related design on the right. We observe a significant decrease in decoding accuracy from good to bad exemplars in both designs. Error bars are SEM over eight subjects. ***p<0.001.

Neural activity patterns were assembled into a sequence of blocks with fixation periods (zero activity) closely mirroring our experimental design. The neural activity was then convolved with a realistic hemodynamic response function (HRF), and normally distributed measurement noise was added. The variance of the measurement noise was estimated from the residuals of the univariate regression analysis of the experimental data. We then performed the same LORO cross validation analysis that we performed on the experimental data. The simulation was repeated 100 times with the PPA voxel activity from each of the eight subjects to estimate the category distribution means. Just as in our fMRI experiment, we found significantly higher decoding accuracy for good (low variance) than bad (high variance) exemplars (t(7) = 20.9, p<0.001, two-tailed, paired t test; Figure 8).

We chose a block design for our experiment because of its more robust signal compared to event-related designs [27]. Would we expect to see a similar pattern of decoding accuracy if we had used an event-related design? To make the comparison we also simulated an event-related experiment with comparable total scan time and the same number of image exemplars. We found the same effect of higher decoding accuracy for good than bad exemplars as in the block design (t(7) = 10.8, p<0.001 two-tailed, paired t test; fig. 8), although decoding accuracy overall was lower for the event-related than the block design, validating our design choice.

Overall, these results suggest that the higher variance among bad compared to good exemplars may account for the difference in decoding that we find. Furthermore, this would be the case regardless of the design we used. Indeed, variance is an issue whenever one is creating a category (training) or assessing membership (testing), because it means that any one exemplar is a less reliable predictor of either the mean (i.e. a prototype) or boundaries of the category. This is certainly true of our classification analysis, but we note that the human brain could also suffer from the same problem. In other words, participants may rate images that share some but not all features of the majority of members of a category as bad, resulting in a set of images whose physical attributes vary more widely than the set of attributes that more clearly and reliably predict the category.

Finally, we would like to note that although variance may be an issue for the purposes of creating or assessing category membership, the same variability is a benefit in distinguishing between members of a category. For example, memory for a particular beach will be better when the set of possible beaches share fewer attributes [28]–[29]. Similarly, we would predict greater fMRI decoding accuracy in distinguishing *between exemplars* when the set of images are drawn from the bad exemplar than the good exemplar sets. However, because we were interested in the category signal we did not design the experiment in such a way that we could separate out the individual exemplars.

### Univariate fMRI Analysis

In our multivariate analysis we have shown that V1, PPA and RSC contain category-related information such that in these ROIs category is decoded more accurately from good than from bad exemplars of natural scenes. How might these results relate to the mean fMRI signal in these areas?

**Figure 9:**
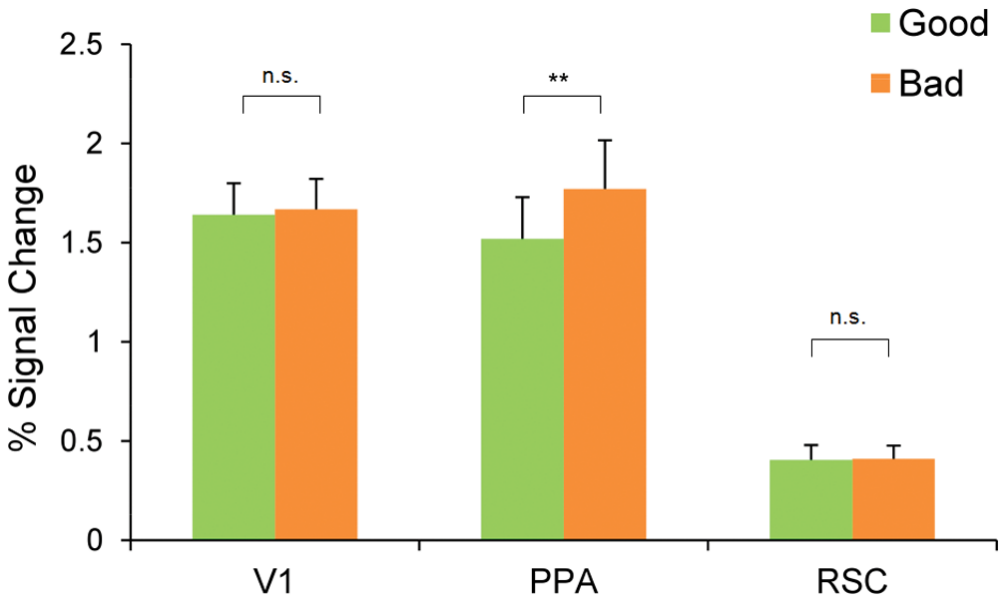
BOLD signal. Percent change in BOLD signal in V1, PPA, and RSC for good (green) and bad (orange) exemplar blocks. **p<0.01.

The univariate analysis revealed that the superior decoding accuracy for good exemplars is not due to a higher BOLD signal for good than bad exemplars. The percent signal change was significantly higher for bad exemplars than good exemplars in PPA (t(7) = 4.34, *p*<0.01; see Figure 9) but failed to reach significance in RSC (t <1) and V1 (t<1). These data suggest that the higher decoding accuracy for good exemplars is due to clearer activity patterns in these ROIs rather than higher overall BOLD signal.

Why might bad exemplars evoke greater BOLD activity in the PPA than good exemplars? One possibility is that the increased variance in the bad exemplars as compared to good exemplars led to this difference. Specifically, because good exemplars are more similar to one another, the good blocks might exhibit stronger repetition suppression (sometimes referred to as “fMRI adaptation”) than the bad [30].

To assess this possibility we performed an additional univariate linear regression where we defined four regressors corresponding to first and second halves (4 time points) of the blocks of good and bad exemplar of natural scenes. These four regressors were modeled separately and convolved by a gamma function to approximate the hemodynamic response [24]. We extracted the percent signal change based on the coefficients associated with these regressors in each participant’s V1, PPA and RSC ROIs. In each ROI mean percent signal change obtained for each of these four conditions was submitted to a 2×2 ANOVA with good versus bad exemplars as one factor and first half versus second half of the block as the other factor. Neither the main effects nor the interaction were significant in V1 (F<1 for the main effect good versus bad, p = 0.099 for the main effect first versus second half, and p = 0.214 for the interaction) and RSC (all Fs<1). However, in PPA, the main effect of the good (1.51 percent signal change) versus bad (1.77 percent signal change) factor was significant (F(7) = 18.95, MSE = 0.027, p<0.01), in keeping with the previous univariate analysis. The main effect of first versus second half of the block also reached significance (F(7) = 5.9, MSE = 0.070, p<0.05). Mean percent signal chance for the first half of the block was significantly higher than for the second half of the block (1.75 and 1.53 respectively), reflecting the fact that the signal diminished over the course of the block. However, the interaction between these two factors did not reach significance (F(7) = 3.06, MSE = 0.012, p = 0.123) indicating that the suppression was similar for good and bad exemplars. In other words, although there may have been repetition suppression over the course of the block, this suppression is not sufficient to explain the overall BOLD difference between good and bad exemplars.

What else might explain the greater activity for bad than good exemplars in the PPA? Our behavioral data show that participants were not only less accurate at categorizing bad exemplars than good exemplars but they were also slower, indicating that they find the bad exemplars harder to categorize than the good exemplars. One possibility then, for the greater BOLD signal in PPA, is that bad exemplars required greater attentional resources than good exemplars of the natural scene categories [31].

## General Discussion

### Better Exemplars, Better Categorization and Better Decoding of Brain Signals

Previous work has shown that natural scene categories are distinguishable in the pattern of activity in V1, PPA, RSC and LOC [5]. In our current study, we asked whether these regions and human subjects were sensitive to the degree to which an image exemplified its category. In a behavioral study we confirmed that good exemplars of our categories were categorized significantly faster and more accurately than bad exemplars. This benefit for good exemplars was present for all six categories. A similar benefit was seen in decoding category from fMRI patterns in visual cortex, specifically PPA, RSC and V1. The difference between good and bad exemplars was assessed by training and testing a decoder on good and bad exemplars separately, and comparing their accuracies. Decoding accuracy was significantly higher in V1, PPA and RSC for good than bad exemplars. This was true despite the fact that there was either no difference in overall BOLD signal evoked by good and bad scenes (RSC and V1), or the signal was actually stronger for bad scenes (PPA). These data not only implicate all three regions in the representation of scene category, but also show that their activity patterns mirror the fundamental graded nature of human categories. Our decoding results also suggest that the differences between good and bad exemplars range from low-level features (decodable in V1) to more complex properties represented in RSC and PPA, such as scene layout [9]–[10].

Since LOC had been previously implicated in natural scene category processing [5], [15], we also explored whether LOC was sensitive to the degree to which an image exemplified its category. In contrast to the earlier study, decoding in LOC did not exceed chance. The current study used a different, although not a wholly disjoint, set of images than Walther et al. [5], and thus the lack of significant decoding in LOC may indicate that the associations of particular objects with a particular scene category were not as consistent (i.e. less diagnostic of scene category) in this image set as that used by Walther et al. [5]. In keeping with this hypothesis, LOC failed to produce above chance decoding in a later study [14] that used a subset of the images used here. We also note that MacEvoy and Epstein [1], who also implicated LOC in scene categorization, used man-made scenes that were readily identified by the presence of diagnostic objects (e.g. a bathtub in a bathroom).

### Why are Good Exemplars Decoded Better than Bad?

One possibility for the decoding advantage in both V1 and later visual areas is that good exemplars are all more similar to a particular prototype than bad exemplars [32]–[33]. Indeed, the pixel-wise average of good and bad exemplars revealed consistency in spatial structure and color among good exemplars of a category. Our analysis of the distribution of good and bad images across the “form” and “color” space further confirmed that the variance in ”form” space among the good examplars was smaller than the variance among the bad examplars, for all six categories. A similar pattern was seen in color space, albeit less consistently across our categories. Of course, such a difference in variance could also explain our decoding results. In fact, our fMRI simulation results show that differential variance among good and bad exemplars of natural scenes leads to similar differences in decoding accuracy as we obtained from our fMRI data. We note, however, that higher variance among bad exemplars could be an intrinsic part of what makes them bad exemplars, contributing to a less clear scene category signal in the brain (in accordance with our fMRI data) and less robust categorization (in accordance with our behavioral data).

That good exemplars of a category are more similar to each other in terms of low-level images statistics is consistent with computational models suggesting that each scene category has a unique “spatial envelop”, which captures scene structure and layout [34]–[35]. However, we note that these results are also consistent with models of categories in which there exists a prototype of each category and good images are more tightly clustered around the prototype than bad images are [32]–[33]. Indeed, our average good images are highly suggestive of a prototype.

Moreover, given the speed with which natural scenes are processed [1]–[2], [4]–[5], [8] it is reasonable to suppose that V1 would be sensitive to differences between prototypes. In other words, the low-level spatial envelope model and organization of a category around a prototype need not be seen as alternative explanations of scene category but instead different level descriptions of the same phenomena: good exemplars are more similar to a prototype than bad, and those features that distinguish between prototypes are computable by even V1.

### Good and Bad Exemplars and Typicality

We are not the first to use representativeness as a tool to better understand the structure of natural scene categories. In particular, others have used typicality ratings to explore the relationship between natural scene categories and global image properties [35] and semantic content models [36]. We note, however, that although our good and bad exemplars presumably bear a close relationship to typicality measures, we did not have our raters rate the images for typicality per se. Instead, we asked them to rate how representative the exemplar was of its category, and included the labels “good” and “poor” at each end of the scale. To the extent that our good and bad exemplars reflect differences in typicality (i.e. the most typical exemplar is the one that shares the highest number of features with the rest of the members of the category [37]), our behavioral results are consistent with previous work on typicality [38].

### Summary

We have shown that the degree to which an image exemplifies a category has consequences for the way participants categorized those scenes and, importantly, for the neural signals they produced. Our decoding results reveal that good exemplars produced clearer and more discriminable patterns of neural activity than bad exemplars of a category. Importantly, this pattern of results is not due to a higher mean signal for good images as PPA, RSC and V1 showed equivalent or lower BOLD activity for good exemplars than for bad. In other words, a more stable pattern of activity appears to underlie the representation of good exemplars of complex scene categories. Our analysis of the images statistics not only reveals that good images produce a more discernible average image than bad, but also that good images in each category are more similar to each other in structure than bad images**.** These data are consistent both with low-level models of scene categorization and models in which a category is organized around a prototype. Finally, our simulation results suggest that the differences in variance between good and bad images, and thus the activity patterns they evoke, may be the cause of the superior decoding for good compared to bad exemplars.
